# Highlighting the most impactful papers in ESC heart failure: 2024–2025

**DOI:** 10.1093/eschf/xvag110

**Published:** 2026-04-17

**Authors:** Jan Biegus, Piotr Ponikowski

**Affiliations:** Department of Cardiology, Clinical Department of Intensive Cardiac Care, Faculty of Medicine, Wroclaw Medical University, Institute of Heart Diseases, Borowska 213, Wroclaw 50-556, Poland; Department of Cardiology, Jan Mikulicz Radecki University Hospital in Wrocław, Borowska 213, Wroclaw 50-556, Poland; Department of Cardiology, Clinical Department of Intensive Cardiac Care, Faculty of Medicine, Wroclaw Medical University, Institute of Heart Diseases, Borowska 213, Wroclaw 50-556, Poland; Department of Cardiology, Jan Mikulicz Radecki University Hospital in Wrocław, Borowska 213, Wroclaw 50-556, Poland

**Keywords:** Heart failure, News, Top-cited papers

## Introduction

In this overview, we highlight the most cited papers published in *ESC Heart Failure* in 2024 and 2025. We need to emphasize that this assessment should be interpreted in the context of an obvious inherent time-lag bias, as earlier-published papers had more time to accumulate citations. The analysis is based on data available as of March 2026. Here, we briefly present the five most-cited papers published each year. In addition, we present five papers published in 2025 that achieved the highest Altmetric Scores as of March 2026, reflecting their broader online visibility and attention. The purpose of this overview is to recognize and celebrate the papers that have made a particularly strong impact on the field, and to thank their authors for their valuable contributions to the journal.

## The most cited papers in 2024

The most-cited papers published in *ESC Heart Failure* in 2024 reflect both the breadth of the field and a strong focus on an integrative, systems-level view of heart failure (HF). The leading article, *2024 update in heart failure*, exemplifies the enduring value of authoritative, practice-oriented reviews that distill a rapidly expanding evidence base into clinically meaningful insights. The paper offers a comprehensive overview of recent advances across the spectrum of HF, including epidemiology, pathophysiology, comorbidities, diagnosis, prognosis, and treatment. Beyond this, it captures important developments in acute HF and cardiogenic shock, as well as progress in cardiomyopathies, device-based therapies, remote monitoring, and transcatheter management of valvular heart disease.^1^

Several highly cited contributions addressed fundamental disease mechanisms. The review *Systemic aging fuels heart failure: Molecular mechanisms and therapeutic avenues* highlights the central role of aging-related pathways—including inflammation, mitochondrial dysfunction, and cellular senescence—in shaping the HF phenotype, while also pointing towards emerging therapeutic targets. The article shows that age-related cardiac dysfunction arises from chronic exposure to multiple stressors, including genomic, epigenetic, oxidative, autophagic, inflammatory, and regenerative disturbances, as well as the accumulation of senescent cells. As physiological reserve declines with age, the capacity to maintain homeostasis becomes progressively impaired, ultimately promoting tissue remodelling, organ dysfunction, and vulnerability to HF.^2^

The study *Association between haemoglobin, albumin, lymphocytes, and platelets and mortality in patients with heart failure* evaluated the prognostic value of the HALP score in hospitalized HF patients (*n* = 730). Higher HALP values were independently associated with outcomes, including 1-month and 1-year death risk. Importantly, HALP showed slightly better predictive performance than PNI, CRP, and even the MAGGIC-HF score, suggesting that this simple composite biomarker may be a practical and easily available prognostic marker in HF.^3^

The paper *Global trends in heart failure from 1990 to 2019: An age-period-cohort analysis from the Global Burden of Disease study* provides an important population-level perspective on the evolving burden of HF worldwide. The authors show that although age-standardized prevalence and disability rates declined over time, the absolute global burden of HF increased markedly, reaching >56 million cases in 2019, largely driven by population growth and aging. Importantly, the study also highlights substantial disparities across socio-demographic strata, sexes, and underlying aetiologies.^4^

The review *Obesity: the perfect storm for heart failure* highlights obesity as a powerful and multifaceted contributor to HF, especially HFpEF. The authors show that excess adiposity promotes HF through a complex interplay of cardiac remodelling, epicardial fat accumulation, microvascular and endothelial dysfunction, metabolic derangements, and chronic inflammation. Importantly, the article also underlines the diagnostic and therapeutic challenges in this population, while pointing to weight loss and emerging disease-modifying therapies as key components of future management.^5^ This paper aligns with the recently proposed adipokine hypothesis of HFpEF development.

## The most cited papers in 2025

The article *SGLT2i reduce arrhythmic events in heart failure patients with cardiac implantable electronic devices* provides intriguing real-world data that SGLT2 inhibitors may have clinically relevant antiarrhythmic effects in HFrEF. In 198 patients with ICD or CRT-D monitored remotely, initiation of SGLT2i therapy was associated with a marked reduction in both atrial and ventricular arrhythmic burden, including fewer episodes of atrial fibrillation, non-sustained ventricular tachycardia, and sustained ventricular tachycardia. These intriguing, hypothesis-generating findings extend the benefits of SGLT2 inhibitors beyond conventional HF outcomes and need confirmation in larger prospective studies.^6^

The paper *Implementation of guideline-directed medical therapy in patients with heart failure with reduced ejection fraction (OPTIMA-HF Registry)* highlights a persistent gap between guideline recommendations and the real-world implementation of GDMT in HFrEF. Although individual drug classes were prescribed at high rates, with approximately 95% of patients receiving beta-blockers and 89% receiving RAASi/ARNI, fewer than half were treated with the full four-drug combination. Importantly, a substantial proportion of untreated patients had no clear contraindications, suggesting therapeutic inertia rather than true clinical limitation. These findings emphasize that the key unmet need in contemporary HF care is not the availability of effective drugs, but their timely and comprehensive implementation, including optimization of combination therapy and dose up-titration.^7^

The study *Frailty determinants in heart failure: Inflammatory markers, cognitive impairment and psychosocial interaction’’* highlights frailty as a multidimensional syndrome in HF, shaped not only by disease severity but also by inflammation, cognitive dysfunction, nutritional status, depression, and treatment adherence. Frail patients had higher CRP and NT-proBNP levels, lower haemoglobin, eGFR, and systolic blood pressure, and were more likely to have malnutrition, cognitive impairment, and depressive symptoms. Importantly, the study emphasizes that frailty in HF results from an interplay of biological and psychosocial factors, underscoring the need for a more integrated, patient-centred approach to risk assessment and management.^8^ Frailty is a common phenomenon in HF and is often under-recognized in clinical practice, despite its important association with prognosis.^9^

The retrospective study *The efficacy and safety of sodium–glucose cotransporter 2 inhibitors in patients aged over 80 years with heart failure* provides important real-world evidence in a highly underrepresented population. In a large cohort (*n* = 1559) of Japanese patients aged ≥80 years, SGLT2 inhibitors were associated with a significant reduction in all-cause mortality and HF hospitalizations, without an increase in adverse events. Notably, these benefits were consistent even among patients with high frailty, low body weight, and poor nutritional status—groups typically excluded from randomized trials. These findings challenge the common reluctance to use SGLT2 inhibitors in very elderly patients and support their broader implementation in routine clinical practice.^10^

The study *Early diuretic response and outcome prediction in ambulatory worsening heart failure: Natriuresis versus diuresis* (a post hoc analysis of the SALT-HF trial [*n* = 167] patients) provides important evidence that not all markers of early decongestive response are equally informative. In patients with ambulatory worsening HF treated with intravenous furosemide, 3-h urinary sodium predicted 30-day adverse outcomes, whereas 3-h diuresis did not. These findings reinforce the concept that natriuresis more closely reflects effective decongestion and renal sodium handling than diuresis alone, and suggest that urinary sodium may be the more clinically meaningful parameter for early risk stratification and tailoring outpatient diuretic therapy.^11^

## Altmetric impact from 2025

Beyond citation-based metrics, Altmetric Scores provide complementary insight into the immediate visibility and broader dissemination of research across digital and public platforms. The most impactful papers from 2025, as reflected by Altmetric attention, highlight topics of high clinical relevance, innovation, and translational potential.

The review on precision medicine in HFpEF achieved the highest Altmetric score among papers published in 2025, underscoring the growing shift towards phenotype-driven and individualized therapeutic strategies in a population characterized by marked heterogeneity.^12^ In parallel, the first-in-human study of a cardiac microcurrent device exemplifies emerging device-based therapies and attracts attention for its novelty and potential to address unmet needs in chronic HF.^13^ Diagnostic innovation is reflected in the study on age-adjusted natriuretic peptide thresholds, which addresses a key limitation of current HF diagnostics and proposes a more tailored approach to community-based detection.^14^ Similarly, the idea of integration of intra-abdominal pressure assessment and point-of-care ultrasound to guide decongestive therapy highlights the increasing role of bedside, physiology-guided management in acute HF (ABDOPOCUS-HF trial) and gathered much attention, as evidenced by Altmetric score.^15^ Finally, the comparative analysis of risk prediction indices emphasizes the ongoing need to refine prognostic tools and improve risk stratification following heart failure hospitalization.^16^

In closing, we would like to sincerely thank all authors who contribute their work to *ESC Heart Failure* and help shape and strengthen the journal’s scientific community.

## Declarations

### Data Availability

No new data were generated or analysed in support of this editorial. All data discussed are derived from previously published studies.

## References

[1] Beghini A, Sammartino AM, Papp Z, von Haehling S, Biegus J, Ponikowski P, et al 2024 update in heart failure. ESC Heart Fail 2025;12:8–42. 10.1002/ehf2.14857 PMC11769673 38806171

[2] Fang Z, Raza U, Song J, Lu J, Yao S, Liu X, et al Systemic aging fuels heart failure: molecular mechanisms and therapeutic avenues. ESC Heart Fail 2025;12:1059–80. 10.1002/ehf2.14947 PMC11911610 39034866

[3] Liu L, Gong B, Wang W, Xu K, Wang K, Song G, et al Association between haemoglobin, albumin, lymphocytes, and platelets and mortality in patients with heart failure. ESC Heart Fail 2024;11:1051–60. 10.1002/ehf2.14662 PMC10966267 38243382

[4] Liu Z, Li Z, Li X, Yan Y, Liu J, Wang J, et al Global trends in heart failure from 1990 to 2019: an age-period-cohort analysis from the Global Burden of Disease study. ESC Heart Fail 2024;11:3264–78. 10.1002/ehf2.14915 PMC11424301 38937863

[5] Lembo M, Strisciuglio T, Fonderico C, Mancusi C, Izzo R, Trimarco V, et al Obesity: the perfect storm for heart failure. ESC Heart Fail 2024;11:1841–60. 10.1002/ehf2.14641 PMC11287355 38491741

[6] Mariani MV, Lavalle C, Palombi M, Pierucci N, Trivigno S, D'Amato A, et al SGLT2i reduce arrhythmic events in heart failure patients with cardiac implantable electronic devices. ESC Heart Fail 2025;12:2125–33. 10.1002/ehf2.15223 PMC12055389 39921334

[7] Paolillo S, Basile C, Marzano F, Bruzzese D, Agostoni P, Mattavelli I, et al Implementation of guideline-directed medical therapy in patients with heart failure with reduced ejection fraction (OpTIMa-HF Registry). ESC Heart Fail 2025;12:1786–95. 10.1002/ehf2.15172 PMC12055350 39909062

[8] Wleklik M, Lee CS, Lewandowski Ł, Czapla M, Jędrzejczyk M, Aldossary H, et al Frailty determinants in heart failure: inflammatory markers, cognitive impairment and psychosocial interaction. ESC Heart Fail 2025;12:2010–22. 10.1002/ehf2.15208 PMC12055405 39853613

[9] Waqas SA, Khan TM, Siddiqi AK, Hassan S, Anker SD. The impact of frailty on in-hospital outcomes in patients with HFrEF. Global Cardiology 2025;3:213–20. 10.4081/cardio.2025.88

[10] Nakano K, Kanenawa K, Isotani A, Morinaga T, Yamamoto K, Miyawaki N, et al The efficacy and safety of sodium-glucose cotransporter 2 inhibitors in patients aged over 80 years with heart failure. ESC Heart Fail 2025;12:2087–97. 10.1002/ehf2.15218 PMC12055332 39829330

[11] Cobo Marcos M, de la Espriella R, Zegri-Reiriz I, Llacer P, Rubio Gracia J, Comín-Colet J, et al Early diuretic response and outcome prediction in ambulatory worsening heart failure: natriuresis versus diuresis. ESC Heart Fail 2025;12:2460–6. 10.1002/ehf2.15275 PMC12287789 40152188

[12] Rasalam R, Sindone A, Deed G, Audehm RG, Atherton JJ. State of precision medicine for heart failure with preserved ejection fraction in a new therapeutic age. ESC Heart Fail 2025;12:1544–57. 10.1002/ehf2.15205 PMC12055434 39844745

[13] Kosevic DB, Radak U, Vukovic P, Schmitto JD, Brandes K, Goettel P, et al Two-year outcomes of a cardiac microcurrent device in chronic heart failure: a first-in-human pilot study. ESC Heart Fail 2025;12:3264–75. 10.1002/ehf2.15369 PMC12450836 40634236

[14] Taylor CJ, Taylor KS, Jones NR, Ordóñez-Mena JM, Bayes-Genis A, Hobbs FDR. Age-adjusted natriuretic peptide thresholds for a diagnosis of heart failure in the community: diagnostic accuracy study. ESC Heart Fail 2025;12:3552–68. 10.1002/ehf2.15383 PMC12450762 40717271

[15] Josa-Laorden C, Campos-Saenz de Santamaría A, Crespo-Aznarez S, Pérez-Silvestre J, Montero-Hernandez E, Llacer-Iborra P, et al The role of intra-abdominal pressure and point of care ultrasound to guide decongestive therapies in acute heart failure. ESC Heart Fail 2025;12:3741–9. 10.1002/ehf2.15380 PMC12450796 40739778

[16] Averbuch T, Zafari A, Islam S, Lee SF, Sankaranarayanan R, Greene SJ, et al Comparative performance of risk prediction indices for mortality or readmission following heart failure hospitalization. ESC Heart Fail 2025;12:1227–36. 10.1002/ehf2.15129 PMC11911581 39835342

